# EHMTI-0169. Trajectories of headache days over one year (5 waves) in chronic and episodic migraineurs participating in the chronic migraine epidemiology and outcomes (cameo) study

**DOI:** 10.1186/1129-2377-15-S1-D4

**Published:** 2014-09-18

**Authors:** RB Lipton, D Serrano, AM Adams, DC Buse, AI Scher

**Affiliations:** 1 The Saul R. Korey Department of Neurology, Albert Einstein College of Medicine and Montefiore Headache Center, Bronx, USA; 2 Biostatistics, Vedanta Research, Chapel Hill, USA; 3 Global Medical Affairs, Allergan Inc., Irvine, USA; 4 Department of Preventative Medicine and Biometrics, Uniformed Services University of the Health Sciences, Bethesda, USA

## Introduction

Within-person variation in headache (HA) frequency and the nature and predictors of this variation have rarely been studied.

## Aims

To determine variation and predictors of HA days/month in persons with episodic (EM) or chronic migraine (CM).

## Methods

CaMEO was a longitudinal US survey employing quota sampling for screening 80,783 respondents to identify persons with migraine. Baseline HA day frequency was classified as EM (modified ICHD-3b migraine diagnosis, <15 HA days/month for past 3 months) or CM (modified ICHD-3b diagnosis, ≥15 HA days/month for past 3 months). Migraineurs completed quarterly surveys over 5 waves (~1 year) assessing HA symptoms and frequency. Using repeated measures regression modeling, we describe variation in HA days within/between individuals, and evaluate potential variation predictors.

## Results

From 58,418 useable returns, we identified 16,789 migraineurs at baseline (EM: 15,313 [91.2%], CM: 1,476 [8.8%]). The reported number of HA days/month showed cyclic variation over time within and between respondents with both CM and EM. HA days increased more for CM (vs EM) persons over time (quarterly RR=1.26, 95% CI 1.20–1.33; P<0.0001). A comprehensive graphical decomposition of this interaction will be presented.

## Conclusion

Substantial within- and between-person heterogeneity in quarterly estimates of HA days/month was observed, and resulting trajectories differed between groups. The number of HA days/month increased 26% more per quarter for persons with CM vs EM, possibly resulting from a lowered threshold for migraine initiation, creating positive feedback leading to more attacks.

## Funding

Allergan

